# Parenteral artemisinins are associated with reduced mortality and neurologic deficits and improved long-term behavioral outcomes in children with severe malaria

**DOI:** 10.1186/s12916-021-02033-1

**Published:** 2021-07-28

**Authors:** Andrea L. Conroy, Robert O. Opoka, Paul Bangirana, Ruth Namazzi, Allen E. Okullo, Michael K. Georgieff, Sarah Cusick, Richard Idro, John M. Ssenkusu, Chandy C. John

**Affiliations:** 1 Ryan White Center for Pediatric Infectious Disease and Global Health, Indiana University School of Medicine, R4 402C 1044 West Walnut St, Indianapolis, IN 46202 USA; 2 Department of Paediatrics and Child Health, Makerere University College of Health Sciences, Kampala, Uganda; 3 Department of Psychiatry, Makerere University College of Health Sciences, Kampala, Uganda; 4 Clinical Epidemiology Unit, Makerere University College of Health Sciences, Kampala, Uganda; 5 Department of Pediatrics, University of Minnesota, Minneapolis, MN USA; 6 Department of Epidemiology and Biostatistics, Makerere University School of Public Health, Kampala, Uganda; 7 Division of Global Pediatrics, University of Minnesota Medical School, Minneapolis, USA

**Keywords:** Artemisinin, Artesunate, Quinine, Mortality, Severe malaria, Neurologic deficit, Long-term, Inflammation, Behavior, Hospital readmission, Pediatric, Children, Cerebral malaria, Severe anemia

## Abstract

**Background:**

In 2011, the World Health Organization recommended injectable artesunate as the first-line therapy for severe malaria (SM) due to its superiority in reducing mortality compared to quinine. There are limited data on long-term clinical and neurobehavioral outcomes after artemisinin use for treatment of SM.

**Methods:**

From 2008 to 2013, 502 Ugandan children with two common forms of SM, cerebral malaria and severe malarial anemia, were enrolled in a prospective observational study assessing long-term neurobehavioral and cognitive outcomes following SM. Children were evaluated a week after hospital discharge, and 6, 12, and 24 months of follow-up, and returned to hospital for any illness. In this study, we evaluated the impact of artemisinin derivatives on survival, post-discharge hospital readmission or death, and neurocognitive and behavioral outcomes over 2 years of follow-up.

**Results:**

346 children received quinine and 156 received parenteral artemisinin therapy (artemether or artesunate). After adjustment for disease severity, artemisinin derivatives were associated with a 78% reduction in in-hospital mortality (adjusted odds ratio, 0.22; 95% CI, 0.07–0.67). Among cerebral malaria survivors, children treated with artemisinin derivatives also had reduced neurologic deficits at discharge (quinine, 41.7%; artemisinin derivatives, 23.7%, *p*=0.007). Over a 2-year follow-up, artemisinin derivatives as compared to quinine were associated with better adjusted scores (negative scores better) in internalizing behavior and executive function in children irrespective of the age at severe malaria episode. After adjusting for multiple comparisons, artemisinin derivatives were associated with better adjusted scores in behavior and executive function in children <6 years of age at severe malaria exposure following adjustment for child age, sex, socioeconomic status, enrichment in the home environment, and the incidence of hospitalizations over follow-up. Children receiving artesunate had the greatest reduction in mortality and benefit in behavioral outcomes and had reduced inflammation at 1-month follow-up compared to children treated with quinine.

**Conclusions:**

Treatment of severe malaria with artemisinin derivatives, particularly artesunate, results in reduced in-hospital mortality and neurologic deficits in children of all ages, reduced inflammation following recovery, and better long-term behavioral outcomes. These findings suggest artesunate has long-term beneficial effects in children surviving severe malaria.

**Supplementary Information:**

The online version contains supplementary material available at 10.1186/s12916-021-02033-1.

## Background

Malaria remains an important cause of morbidity and mortality in children, with an estimated 272,000 global malaria deaths in children under 5 years of age in 2018 [1]. Two common manifestations of pediatric severe malaria (SM) in Ugandan children are cerebral malaria (CM) and severe malarial anemia (SMA). CM is associated with high mortality (18-21%) [2], with children at risk of neurologic sequelae, and problems in cognition, attention, memory, and behavior [3–10]. Children with SMA are at increased risk of post-discharge morbidity including repeated hospitalizations [11, 12], reduced cognitive functioning, and more behavioral problems compared to community children [8, 9].

In 2011, the World Health Organization recommended intravenous artesunate as the first-line therapy for SM after clinical trials demonstrated superiority of artesunate to quinine to improve parasite clearance and reduce mortality [2, 13–19]. However, these studies mainly evaluated inpatient outcomes and there are limited post-discharge data comparing long-term clinical, cognitive, and behavioral outcomes after treatment with artesunate or quinine.

The objective of this study was to evaluate mortality and long-term outcomes following treatment with quinine or artemisinin derivatives in a prospective cohort study of children with SM that coincided with the roll-out of artesunate as the first-line therapy for SM in Uganda. This post hoc analysis evaluates the impact of artemisinin derivatives on long-term clinical, cognitive, and behavioral outcomes in children with severe malaria.

## Methods

### Study participants

The study was performed at the Mulago National Referral Hospital in Kampala, Uganda, from 2008 to 2015, enrolling children 18 months to 12 years of age [9]. Kampala is an area of relatively low malaria transmission intensity with two seasonal peaks every year [20].

All children with severe malaria had *Plasmodium falciparum* on blood smear. Children with cerebral malaria (CM) had a Blantyre Coma Score <3 with no other identifiable cause: ruling out meningitis, a prolonged postictal state, or hypoglycemia-associated coma reversed by a glucose infusion. Children with severe malarial anemia (SMA) had a hemoglobin level ≤5g/dL. Children with CM and SMA were classified as CM. Age-matched community children (CC) were recruited from the nuclear family, extended family, or neighborhood households of children with severe malaria. Exclusion criteria included prior coma, head trauma, hospitalization for malnutrition, cerebral palsy, or known chronic illness requiring medical care or causing developmental delay. The present study focuses on the children with CM or SMA.

Children were managed according to the Uganda Clinical Guidelines at the time of the study. In the early phase of the study, intravenous quinine hydrochloride was the first-line treatment followed by oral quinine to complete 7 days of treatment. Artemisinin derivatives (artesunate or artemether) were the second-line therapy to be used if quinine was contraindicated or not available. In November 2012, updated Ugandan Clinical Guidelines were published recommending parenteral artesunate as the first-line antimalarial for severe malaria, followed by oral artemether-lumefantrine. The change to artemisinin derivatives was implemented gradually in the health units depending on medication availability and clinician discretion.

Children underwent a medical history and physical examination on enrollment. Caregivers were asked to bring their children back to study hospitals whenever the children fell sick during the follow-up period, and deaths during the study period were also recorded.

In 2010, a nested randomized controlled trial recruited from within the study cohort and children were randomized to delayed or immediate iron therapy and were under active and passive surveillance for illness [21]. Trial details are included in Additional file 1: Methods.

### Laboratory assessment

Peripheral blood smears were used to quantify parasite density using Giemsa staining with standard protocols. EDTA anticoagulated plasma was collected at admission and stored at −80°C until testing. Plasma *P. falciparum* histidine-rich protein-2 (PfHRP2) levels were measured to assess parasite biomass (Cellabs, Australia) [22]. Plasma concentrations of C-reactive protein (CRP) were measured by Luminex immunoassay (Milliplex MAP kit, EMD Millipore, Billerica, MA).

### Neurologic, cognitive, and behavioral assessment

A neurologic deficit at discharge was defined as the presence of motor deficits, ataxia, movement disorder, or behavior, speech or visual disorders in a child with no known prior deficits. Children had neurologic, cognitive, and behavioral assessments a week after discharge and then 6, 12, and 24 months after enrollment. Neuropsychology testers were blinded to the study group. All tests have been adapted for use in Uganda and selected based on earlier studies showing an impact of cerebral malaria on cognition, attention, and memory in Ugandan children 5 to 12 years of age [6, 7]. For the present study, tests were added to assess the same domains in children below 5 years of age. Parental ratings of behavior and executive function were added to examine these outcomes based on a case series reporting behavioral problems in Ugandan children following cerebral malaria [23].

In children <5 years of age, the Mullen Scales of Early Learning (MSEL) [24], Color Object Association Test (COAT) [25], and Early Childhood Vigilance Test (ECVT) [26] were used to assess cognition, associative memory, and attention, respectively, as described [9]. Test details are described in Additional file 1: Methods. In children ≥5 years of age, the Kaufman Assessment Battery for Children (K-ABC second edition) was used to measure overall cognitive ability and memory [27] while the Test of Variables of Attention (TOVA) was used to assess attention [28]. Socio-emotional function was assessed using the preschool (18 months–6 years) and school-aged (6–12 years) Child Behavioral Checklist (CBCL) [29], and executive function with the preschool (2–6 years) and school-aged (6–12 years) Behavior Rating Inventory of Executive Function (BRIEF) [30]. Higher z scores on CBCL and BRIEF indicate poorer performance and more problematic behavior, while higher z scores in cognition, attention, and memory indicate better performance. Age-adjusted z scores were created using the scores of the community children (CC) [9]. Age-adjusted z scores for cognition, attention, memory, and behavior (socio-emotional function and executive function) were generated separately for children stratified by age (<5 or ≥5 for cognition, attention, memory; <6 or ≥6 for behavior, based on test-specified age cutoffs).

### Statistical analyses

Analyses were done using Stata v14.0 (StataCorp. 2015). Differences in continuous variables were assessed using Student’s t test or Wilcoxon rank sum test, as appropriate. Differences in proportions were compared using Pearson’s χ^2^ or Fisher’s exact test. Logistic regression was used to assess the relationship between antimalarial therapy and dichotomous outcomes (neurologic deficit, mortality, death, or readmission in follow-up) and covariates were adjusted based on a known or hypothesized relationship with the outcome.

Primary study outcomes were defined a priori as risk of readmission or death within 6 months of follow-up and z scores for cognitive (overall cognition, attention, memory) and behavioral outcomes (internalizing, externalizing, or total behavior problems) or executive function (global executive composite) over the full 24-month follow-up. A 6-month follow-up was chosen for readmission or death evaluation because most readmissions or deaths occurred during this period (64% of total readmissions or deaths), and because anti-malarial treatment was most likely to relate to this risk within the first 6 months after discharge. Longitudinal changes in cognition, attention, memory, or behavior by antimalarial treatment were assessed using linear mixed effects models including the age-adjusted z scores including a subject specific random intercept, time as a categorical variable to allow for non-linearity between study visits (2 weeks post-hospital discharge, and at 6, 12, and 24 months of follow-up). Models included child age, sex, weight-for-age and height-for-age z scores, socioeconomic status, home environment z score, disease severity during the acute illness (number of seizures, presence of coma, acute kidney injury), child schooling, enrollment in the iron study (not enrolled, enrolled and randomized to immediate iron, enrolled and randomized to delayed iron), and the number of hospital admissions over follow-up as fixed effects. Behavioral outcomes were assessed using a caregiver questionnaire and included a random caretaker effect in behavioral analyses in children <6 years of age. To adjust for multiple comparisons, the Benjamini-Hoch correction for false discovery rate (FDR) was used at 0.05 for each age strata.

### Role of the funding source

The funders had no role in the study design, analysis, or decision to publish.

## Results

### Description of antimalarial use in the study population

**Fig. 1:**
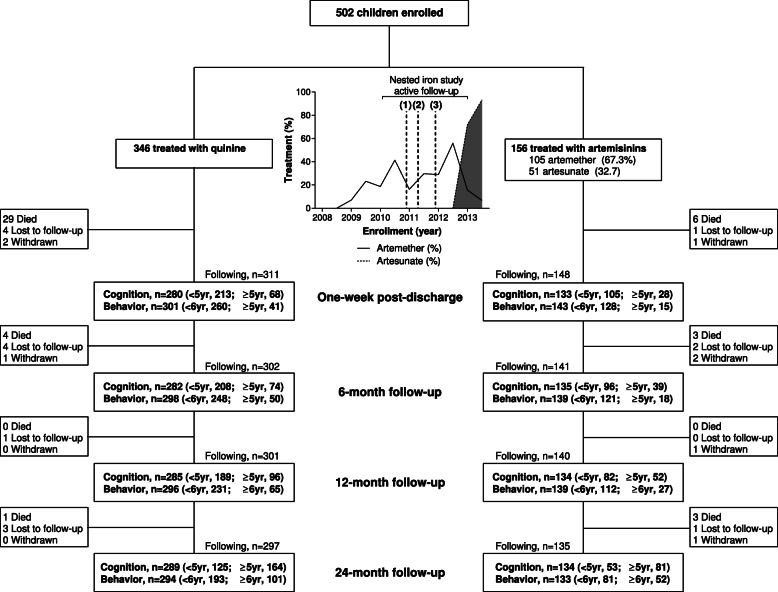
Flow chart of the study population and roll out of parenteral artemisinin derivatives over the study period. Flow chart showing the number of children followed up for cognitive (cognition, attention, memory) and behavioral evaluation at each study time point (1 week following hospital discharge, and 6, 12, and 24 months of follow-up). The number of children who died were lost to follow-up or withdrawn from the study are indicated at each time point. The number of children followed at each time point is indicated, and the difference between the number followed and number tested refers to missed visits or tests not administered. The graph presents the proportion of children treated with parenteral artemisinin over study enrollment with data calculated on a biannual basis (January–June; July–December) from the start of study enrollment in November 2008 until the end of the study in December 2013. Major changes in antimalarial treatment policies are indicated by dashed vertical lines: (1) AQUAMAT results disseminated showing superiority of artesunate over quinine in reducing mortality in African children with severe malaria, November 2010; (2) World Health Organization recommendation of injectable artesunate as the first-line therapy for severe malaria, April 2011; and (3) Ugandan updated clinical guidelines recommending artesunate as the first-line therapy for severe malaria, November 2012

Five hundred two children with SM were enrolled: 269 with CM and 233 with SMA. See Fig. 1 for a flow chart of the study population. Enrollment for this study (2008–2013) coincided with a transition from use of quinine to artesunate for the treatment of SM. Parenteral (intramuscular) artemether was first used in 2009 and intravenous artesunate started being used in 2013 following a change in Ugandan national guidelines recommending artesunate as the first-line therapy for SM in November 2012 (Fig. 1). The nested randomized controlled trial of immediate vs. delayed iron supplementation enrolled children between 2010 and 2013 with one third of the study population enrolled in the nested iron study.

Baseline characteristics of study participants are shown in Table 1. Overall, 68.9% (n=346) of children received intravenous quinine alone, and 31.1% received various combinations of artemisinin therapies including 14.7% (*n*=74) intramuscular artemether and quinine, 6.2% (*n*=31) intramuscular artemether alone, and 10.2% (*n*=51) intravenous artesunate. Differences in clinical factors among children receiving artemether vs. artesunate are outlined in Additional file 1: Table S1. Overall, children receiving artemisinin derivatives for treatment of severe malaria had higher median levels of lactate, lactate dehydrogenase, total bilirubin, and parasite biomass (plasma HRP-2) compared to children receiving quinine alone (*p*<0.05).

**Table 1:** Characteristics of severe malaria study participants by choice of in-hospital antimalarial administered

	Antimalarial treatment		
	Quinine (***n***=346)	Artemsinin derivatives (***n***=156)	P
Age, years	3.7 (1.9)	3.7 (1.7)	0.88
Sex, no. % female	142 (41.0)	61 (39.1)	0.68
Weight-for-age z score	−1.2 (1.1)	−1.0 (1.1)	0.21
Height-for-age z score	−1.2 (1.4)	−1.4 (1.4)	0.20
Duration of fever, history	3.8 (2.7)	3.8 (2.2)	0.96
Socioeconomic status score ^a^	9.4 (3.0)	9.8 (3.4)	0.19
Home environment z score ^a^	−0.10 (0.98)	0.12 (1.02)	0.03
Maternal education, No. (%) ^a^			
Primary 6 or lower	121 (38.2)	55 (36.9)	0.25
Primary 7	69 (21.8)	27 (18.1)	
Secondary or higher	107 (33.8)	62 (41.6)	
Not known	20 (6.3)	5 (3.4)	
Paternal education, No. (%) ^a^			
Primary 6 or lower	61 (19.2)	35 (23.5)	0.02
Primary 7	50 (15.8)	25 (16.8)	
Secondary or higher	130 (41.0)	72 (48.3)	
Not known	76 (24.0)	17 (11.4)	
Child any education, No. (%) ^a^	92 (30.1)	52 (35.6)	0.24
Severe malaria group, No. (%)			
Cerebral malaria	186 (53.8)	83 (53.2)	0.91
Severe malarial anemia	160 (46.2)	73 (46.8)	
Deep breathing, No. (%)	20 (5.8)	20 (12.8)	0.007
**Laboratory characteristics**			
Hemoglobin, g/dL	4.8 (3.9, 7.2)	4.7 (3.6, 6.4)	0.16
Glucose, mmol/L	6.3 (4.8, 8.5)	6.7 (4.8, 8.7)	0.60
Lactate, mmol/L	3.8 (2.2, 6.6)	5.2 (3.1, 8.2)	0.0001
WBC, x10^3^/μL	10.2 (7.3, 15.1)	10.8 (7.9, 15.8)	0.39
Platelet, x10^3^/μL	103 (51, 176)	86 (39, 158)	0.08
Lactate dehydrogenase, U/L	762 (608, 1058)	846 (680, 1153)	0.004
Parasite density, parasites/uL	45280 (11400, 209460)	34485 (9640, 161360)	0.15
Plasma PfHRP2, ng/mL	1654 (563, 3638)	2663 (609, 5671)	0.006
Creatinine, mg/dL	0.38 (0.30, 0.49)	0.38 (0.28, 0.52)	0.79
BUN, mg/dL	14 (10, 21)	16 (11, 23)	0.17
**Clinical complications and recovery**			
Parasite clearance time, days	2 (1, 3)	2 (1, 3)	0.27
Coma duration^b^, hours	48 (29, 80)	56 (41, 85)	0.10
Seizure number in hospital	1 (0, 2)	1 (0, 2)	0.24
Hypoglycemia, No. (%)	26 (7.5)	10 (6.4)	0.66
**Co-treatments**			
Dextrose bolus, No. (%)	203 (58.7)	89 (57.1)	0.73
Transfusion, No. (%)	254 (73.4)	133 (85.3)	0.003
IV fluids, No. (%)	52 (15.0)	26 (16.7)	0.64
Furosemide, No. (%)	71 (20.5)	16 (10.3)	0.005
Antibiotics, No. (%)	174 (50.3)	90 (57.7)	0.12
Enrolled in iron study, No. (%)	77 (22.3)	79 (50.6)	<0.001
Immediate iron, No. (%)	36 (10.4)	42 (26.9)	0.42
Delayed iron, No. (%)	41 (11.9)	37 (23.7)	

Data presented as median (IQR) unless otherwise indicated

^a^Data available for surviving children who had a home visit

^b^Assessed only in children with coma

Children receiving artemisinin derivatives were more likely to have received a transfusion and less likely to receive furosemide than children treated with quinine (*p*<0.05, Table 1).

### Morbidity and mortality associated with parenteral artemisinin derivatives

Overall, inpatient mortality was 8.4% in children receiving quinine compared to 3.9% in children receiving artemisinin derivatives corresponding to an unadjusted odds ratio (OR) of 0.51 (95% CI 0.22, 0.10)  (Table 2). Following adjustment for age, sex, measures of disease severity, and enrollment in the iron study, children treated with artemisinin derivatives had reduced inpatient death compared to children treated with quinine (adjusted OR 0.22 (95% CI 0.07, 0.67) (Table 2).

**Table 2:** Relationship between artemisinin derivatives and mortality, neurologic deficits at discharge, and death or readmission in follow-up

	In-hospital mortality ^a^			Neurologic deficit at discharge in children with cerebral malaria^b^			Death or readmission 6 months of follow-up ^c^		
	OR (95% CI)	aOR (95% CI)	P	OR (95% CI)	aOR (95% CI)	P	OR (95% CI)	aOR (95% CI)	P
Antimalarial class									
Quinine	Reference	Reference		Reference	References		Reference	Reference	
Artemisinin	0.51 (0.22, 1.20)	0.22 (0.07, 0.67)	0.008	0.44 (0.24, 0.81)	0.28 (0.13, 0.59)	0.0007	2.09 (1.22, 3.58)	1.24 (0.68, 2.27)	0.49
Antimalarial medication									
Quinine	Reference	Reference		Reference	Reference		Reference	Reference	
Quinine + Artemether	0.62 (0.21, 1.83)	0.28 (0.08, 0.97)	0.05	0.29 (0.13, 0.67)	0.18 (0.07, 0.48)	0.0005	1.88 (0.93, 3.78)	1.47 (0.68, 3.17)	0.33
Artemether	0.36 (0.05, 2.77)	0.12 (0.01, 1.49)	0.10	0.79 (0.22, 2.82)	0.50 (0.12, 2.13)	0.35	5.49 (2.44, 12.4)	3.88 (1.56, 9.69)	0.004
Artesunate	0.45 (0.10, 1.93)	0.17 (0.02, 1.55)	0.12	0.69 (0.25, 1.94)	0.51 (0.14, 1.83)	0.30	0.96 (0.36, 2.58)	0.30 (0.10, 0.90)	0.03

^a^Adjusted for age, sex, disease group (cerebral malaria or severe malarial anemia), respiratory distress, acute kidney injury, hemoglobin, log base 10 transformed lactate dehydrogenase and total bilirubin concentrations on admission, and enrollment in the iron study (not enrolled, enrolled in the immediate iron arm, enrolled in the delayed iron arm)

^b^Adjusted for age, sex, duration of coma, number of seizures during hospitalization, acute kidney injury, and enrollment in the iron study (not enrolled, enrolled in the immediate iron arm, enrolled in the delayed iron arm)

^c^Adjusted for age, sex, disease group (cerebral malaria or severe malarial anemia), hemoglobin on admission, receiving a transfusion in-hospital, and enrollment in the iron study (not enrolled, enrolled in the immediate iron arm, enrolled in the delayed iron arm)

To evaluate post-discharge morbidity and mortality, we considered a composite endpoint of all-cause mortality or readmission during the first 6 months of follow-up. There was no difference in risk of readmission or death between children with severe malaria treated with quinine vs. artemisinin derivatives. However, looking at individual drug treatments, children treated with artemether alone had a 3.88-fold increased odds of death or readmission in follow-up (95% CI, 1.56 to 9.69) compared to children treated with quinine while children treated with artesunate had a 70% reduced odds of death or readmission (95% CI, 0.10 to 0.90) following adjustment for age, sex, measures of disease severity, and enrollment in the iron study (Table 2).

### Neurologic deficits in severe malaria survivors at discharge and follow-up

Among CM survivors, 83/232 children (35.8%) had neurologic deficits at discharge. Most neurologic deficits recovered by 6-month follow-up with 12 children (4.5%) having persistent neurologic deficits at 6-month follow-up, and 7 children (2.9%) having persistent neurologic deficits at 12 and 24-month follow-up.

The frequency of neurologic deficits at discharge among children receiving artemisinin derivatives was 24.0% compared to 41.9% in children receiving quinine (p=0.008). Following adjustment for age, sex, coma duration, acute kidney injury on admission, and enrollment in the iron study, children receiving artemisinin derivatives had an aOR for neurologic deficits of 0.28 (95% CI, 0.13 to 0.59) (Table 2). By 6-month follow-up, there were no differences in the frequency of neurologic deficits based on antimalarial therapy.

### Cognitive and behavioral outcomes in children based on antimalarial treatment

There were no differences in cognition, attention, or memory in either preschool or school aged children based on anti-malarial treatment (quinine vs. artemisinin derivatives) (Table 3). Preschool children treated with artemisinin derivatives showed evidence of improved internalizing, externalizing, and total behavior using the Child Behavior Checklist (CBCL) and had improved executive function using the Behavior Rating Inventory of Executive function (BRIEF) (adjusted p value <0.05, Table 3). Effects were largest in children receiving artesunate (Table 4) and were consistent across study groups with improved outcomes in children with cerebral malaria and severe malarial anemia (Additional file 1: Table S2 and Table S3).

**Table 3:** Primary cognitive and behavioral outcomes in children over 24-month follow-up, according to antimalarial treatment

	Preschool age at testing				School age at testing			
	N (obs), N ^**a**^	Mean difference^**b**^, artemisinin derivatives vs. quinine (95% CI)	*P* value	Sig.†*	N (obs), N ^**a**^	Mean difference^**b**^, artemisinin derivatives vs. quinine (95% CI)	*P* value	Sig.†*
**Cognition outcomes** ^**c**^	**<5 years of age**				**≥ 5 years of age**			
Cognition	1053, 351	-0.04 (-0.37, 0.29)	0.83		584, 248	-0.05 (-0.51, 0.41)	0.84	
Attention	1111, 352	0.06 (-0.14, 0.26)	0.55		590, 251	0.19 (-0.18, 0.56)	0.31	
Memory	1091, 350	-0.09 (-0.23, 0.06)	0.23		594, 251	0.13 (-0.27, 0.53)	0.53	
**Behavioral outcomes** ^**d**^	**<6 years of age**				**≥ 6 years of age**			
**Child Behavior Checklist**								
Internalizing behavior	1339, 390	-0.38 (-0.57, -0.18)	0.0002	†*	353, 152	-0.33 (-0.64, -0.02)	0.039	†
Externalizing behavior	1339, 390	-0.37 (-0.56, -0.27)	0.0002	†*	353, 152	-0.36 (-0.84, 0.12)	0.145	
Total behavior	1339, 390	-0.42 (-0.64, -0.21)	0.0001	†*	353, 152	-0.44 (-0.89, 0.02)	0.060	
**Behavior rating inventory**								
Global Executive Composite	735, 306	-0.87 (-1.24, -0.51)	<0.0001	†*	247, 139	-0.45 (-0.80, -0.10)	0.012	†

^a^N (obs) refers to the number of observations of cognitive or behavioral measures included in the model and N refers to the number of study participants

^b^Mean difference and 95% confidence interval (CI) are derived from a linear mixed effects model, with the beta coefficient representing the mean difference. Models included a subject-specific random intercept and random caretaker effect, time as a categorical variable (to allow for non-linearity between study visits), and adjusted for age, sex, height-for-age and weight-for-age z score, socioeconomic status and home environment z score, disease severity during the acute illness (number of seizures, coma, acute kidney injury), child schooling, enrollment in the iron study (not enrolled, enrolled in the immediate iron arm, enrolled in the delayed iron arm), and the number of hospital admissions over 24 months follow-up

^c^For cognitive outcomes a positive number is indicative of an improved outcome in children receiving artemisinin derivatives. In preschool-aged children (<5 years of age), cognition was assessed using the Mullen Scales of Early Learning, attention was assessed using the early childhood vigilance test, and memory was assessed using the color object association test. In school aged children (≥5 years of age), cognition was assessed using the Kauffman Assessment Battery for Children (K-ABC) second edition using the mental processing index, attention was assessed using the Test of Variables of Attention using the D-prime measure, and memory was assessed using the sequential processing score from the K-ABC

^d^For behavioral outcomes, a negative number is indicative of an improved outcome in children receiving artemisinin derivatives

†p<0.05, *adjusted p<0.05 Benjamini-Hoch correction for false discovery rate at 0.05 adjusting for 7 comparisons within each age strata

**Table 4:** Primary cognitive and behavioral outcomes in children over 24- month follow-up, according to a specific antimalarial drug treatment

	Preschool age at testing				School age at testing			
	N (obs), N^a^	Mean difference^b^, artemisinin derivative vs. quinine (95% CI)	*P* value	Sig. †*	N (obs), N^a^	Mean difference^b^, artemisinin derivative vs. quinine (95% CI)	*P* value	Sig. †*
**Cognition outcomes**^**c**^	**<5 years of age**				**≥ 5 years of age**			
Cognition								
Quinine + Artemether	1053, 351	−0.10 (−0.50, 0.30)	0.618		584, 248	−0.03 (−0.63, 0.58)	0.928	
Artemether		0.38 (−0.25, 1.01)	0.234			0.33 (−0.49, 1.15)	0.431	
Artesunate		−0.17 (−0.70, 0.36)	0.531			−0.36 (−1.06, 0.35)	0.319	
Attention								
Quinine + Artemether	1111, 352	0.09 (−0.15, 0.32)	0.463		590, 251	0.11 (−0.38, 0.60)	0.650	
Artemether		0.04 (−0.36, 0.44)	0.844			0.47 (−0.19, 1.13)	0.159	
Artesunate		0.01 (−0.31, 0.33)	0.968			0.10 (−0.47, 0.67)	0.727	
Memory								
Quinine + Artemether	1091, 350	−0.07 (−0.24, 0.10)	0.426		594, 251	0.14 (−0.39, 0.67)	0.602	
Artemether		0.05 (−0.22, 0.33)	0.715			0.11 (−0.61, 0.82)	0.766	
Artesunate		−0.22 (−0.45, 0.01)	0.060			0.13 (−0.48, 0.74)	0.680	
**Behavioral outcomes**^**d**^	**<6 years of age**				**≥ 6 years of age**			
**Child Behavior Checklist**								
Internalizing behavior								
Quinine + Artemether	1339, 390	−0.16 (−0.39, 0.07)	0.181		353, 152	−0.24 (−0.68, 0.190	0.268	
Artemether		−0.42 (−0.79, −0.05)	0.026	†		−0.08 (−0.62, 0.45)	0.765	
Artesunate		−0.82 (−1.13, −0.51)	<0.0001	†*		−0.64 (−1.11, −0.17)	0.008	†*
Externalizing behavior								
Quinine + Artemether	1339, 390	−0.13 (−0.46, 0.10)	0.261		353, 152	−0.08 (−0.72, 0.56)	0.804	
Artemether		−0.51 (−0.87, −0.16)	0.005	†*		0.26 (−0.52, 1.05)	0.508	
Artesunate		−0.76 (−1.06, −0.46)	<0.0001	†*		−1.24 (−1.95, −0.54)	0.0006	†*
Total behavior								
Quinine + Artemether	1339, 390	−0.21 (−0.46, 0.05)	0.111		353, 152	−0.19 (−0.79, 0.41)	0.527	
Artemether		−0.48 (−0.88, −0.07)	0.021	†		0.23 (−0.51, 0.97)	0.543	
Artesunate		−0.84 (−1.18, −0.50)	<0.0001	†*		−1.32 (−1.97, −0.66)	0.0001	†*
**Behavior rating inventory**								
Global executive composite								
Quinine + Artemether	735, 306	−0.49 (−0.94, −0.05)	0.031	†	247, 139	−0.21 (−0.66, 0.24)	0.361	
Artemether		−0.87 (−1.56, −0.17)	0.0144	†		−0.20 (−0.77, 0.36)	0.480	
Artesunate		−1.48 (−2.00, −0.95)	<0.0001	†*		−1.00 (−1.50, −0.50)	0.0001	†*

^a^N (obs) refers to the number of observations of cognitive or behavioral measures included in the model and N refers to the number of study participants

^b^Mean difference and 95% confidence interval (CI) are derived from a linear mixed effects model, with the beta coefficient representing the mean difference. Models included a subject-specific random intercept and random caretaker effect, time as a categorical variable (to allow for non-linearity between study visits), and adjusted for age, sex, height-for-age and weight-for-age z score, socioeconomic status and home environment z score, disease severity during the acute illness (number of seizures, coma, acute kidney injury), child schooling, enrollment in the iron study (not enrolled, enrolled in the immediate iron arm, enrolled in the delayed iron arm), and the number of hospital admissions over 24 months follow-up

^c^For cognitive outcomes a positive number is indicative of an improved outcome in children receiving artemisinin derivatives. In preschool-aged children (<5 years of age), cognition was assessed using the Mullen Scales of Early Learning, attention was assessed using the early childhood vigilance test, and memory was assessed using the color object association test. In school-aged children (≥5 years of age), cognition was assessed using the Kauffman Assessment Battery for Children (K-ABC) second edition using the mental processing index, attention was assessed using the Test of Variables of Attention using the D-prime measure, and memory was assessed using the sequential processing score from the K-ABC

^d^For behavioral outcomes, a negative number is indicative of an improved outcome in children receiving artemisinin derivatives

†p<0.05, *adjusted p<0.05 Benjamini-Hoch correction for false discovery rate at 0.05

School-age children (≥6 years) treated with artemisinin derivatives had improved internalizing behavior and executive function compared to children treated with quinine, but the effects were not significant after adjustment for multiple comparisons. When looking at individual drug treatments, artesunate was associated with improved behavior and executive function in school-age children (Table 4) and this effect was evident only in children with severe malarial anemia (Additional file 1: Table S3).

### Children treated with parenteral artemisinin derivatives have lower inflammation at a 1-month follow-up

**Fig. 2:**
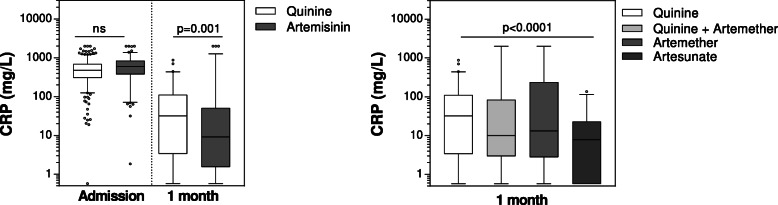
Treatment with artesunate is associated with reduced systemic inflammation at 1-month follow-up. Box and whisker plots showing the median (interquartile range) and 95% confidence interval of plasma CRP levels measured on enrollment and at 1-month follow-up. The p values on the graph are derived from linear regression models adjusting for child age, sex, and treatment group in the iron study

In order to understand whether a more rapid reduction in systemic inflammation could be a mechanism leading to improved behavioral outcomes, we assessed CRP levels at 1-month follow-up in children enrolled in the iron study. Levels of CRP were lower in children treated with artemisinin derivatives compared to children treated with quinine and were lowest in children treated with intravenous artesunate (Fig. 2). Artemisinin derivatives were independently associated with reduced CRP levels at 1-month follow-up relative to children treated with quinine adjusting for age, sex, and iron treatment arm (beta, −1.01 [95% CI, −1.61 to −0.40]; p=0.001). Treatment with artesunate, but not artemether, was associated with reduced CRP levels at follow-up compared to children treated with quinine (artesunate, beta, −1.90 [95% CI, −2.56 to −1.25]; p<0.000; artemether, beta, 0.47 [95% CI, −0.22 to 1.17]; p=0.181).

## Discussion

In the present study, we show that children with severe malaria treated with artemisinin derivatives have lower mortality than children treated with quinine. The unadjusted mortality benefit and confidence intervals associated with artemisinin derivative use compared to quinine in this cohort (OR, 0.51 95% CI 0.22, 1.20) is compatible with estimates from children in Africa treated with artesunate vs. quinine (OR, 0.69 95% CI 0.57, 0.84) [2, 17, 31]. The present study confirms the superior efficacy of artesunate to quinine in the treatment of severe malaria.

We also demonstrate the novel finding that artemisinin derivatives, compared to quinine, are associated with less acute neurologic deficits in children with cerebral malaria and better long-term behavioral and executive function outcomes in preschool children with severe malaria. Early animal studies suggested potential neurotoxicity with artemisinin derivative treatment [32–35]. However, the doses used in these studies were high, and findings of neurotoxicity have not been substantiated in humans. In contrast, newer studies have assessed the anti-inflammatory properties of artesunate in mouse models of traumatic brain injury [36] and experimental cerebral malaria [37] and found it to be neuroprotective in these conditions. Consistent with these animal studies, we found artemisinin derivatives to be associated with fewer neurologic deficits at discharge than quinine in children with CM (41.9% of children treated with quinine had neurologic deficits compared to 24.0% of children treated with artemisinin derivatives, p=0.008) and with improved internalizing and externalizing behaviors and better executive function over 2 years of follow-up in children with CM or SMA. However, overall cognition, attention, and associative memory did not differ by anti-malarial treatment. The differences in behavior and executive function remained significant after adjusting for demographic, socioeconomic risk factors, disease severity, and other factors that could potentially affect these outcomes such as enrollment in the iron sub-study, caregiver answering the questionnaire, and the incidence of hospitalizations over a 24-month follow-up.

In murine studies, artesunate treatment of CM decreased pro-inflammatory cytokine production in the hippocampus [37], reduced astrocyte and microglia activation, down-regulated multiple inflammasome pathways, and modulated neurotropic factors that affect neuronal survival in a model of traumatic brain injury [36], including brain-derived neurotropic factor, which has been implicated in CM severity [38]. We also observed significantly lower levels of CRP at 1-month follow-up in surviving children treated with artemisinin derivatives. CRP is a measure of systemic inflammation, and elevated CRP levels have been associated with worse neurodevelopment in a variety of pediatric populations [39–42]. In order to understand the role of inflammation in malaria-associated neurodevelopment, additional studies are needed to assess markers of inflammation and endothelial activation over clinical recovery to delineate potential pathways of neuroprotection in relation to long-term neurodevelopment and behavioral outcomes. Systemic and sustained inflammatory responses during critical periods of brain development and maturation can lead to disrupted development [43–45], particularly in myelinated pathways in the frontal lobe. Further, systemic inflammation can divert nutrients (e.g., iron, amino acids) that are necessary for brain development [46, 47]. Executive function and behavior are regulated more by the frontal lobe, whereas cognition is regulated more by association areas of the brain such as the temporal, parietal, and occipital intersections. Artesunate could potentially protect executive function and improve behavioral outcomes through one or more of these pathways. Future studies will need to explore potential pathways by which artesunate affects behavior and executive function and whether this is mediated by faster parasite clearance that has been observed in a number of clinical populations, including this one. The salutary effect of artesunate on these functions is an additional long-term benefit from this treatment to survivors of severe malaria.

Overall, there was no difference in the odds of post-discharge readmission or death compared to quinine (OR, 1.24, 95% CI, 0.68 to 2.27) in the first 6 months of follow-up. In sub-group analysis, artemether was associated with an increased risk of readmission or death, and this risk was independent of study group (SMA or CM), being enrolled in the iron study, or receipt of blood transfusion. It is unclear why artemether was associated with increased risk of readmission or death, and as this was sub-group analysis with relatively small numbers, additional study will be required to confirm this association and explore potential mechanisms by which it might occur. We observed a 70% reduced odds of readmission or death in children receiving artesunate within the first 6 months of follow-up (95% CI, 0.10 to 0.90) consistent with the more rapid parasite clearance in this group. Children treated with artesunate had faster parasite clearance than children treated with quinine, consistent with rapid absorption of artesunate, which has broad activity against both ring and mature stages of the parasite life-cycle [48], is water soluble [49], and rapidly reaches therapeutic plasma levels of dihydroartemisinin [50, 51]. Artemether, an oil-based formulation, is administered intramuscularly and has slower and more variable absorption than artesunate [51–53].While artemether may have activity against ring-stage parasites, its slower absorption did not confer the same clearance advantage as artesunate given the frequency of blood sampling in this study and was not associated with the same anti-inflammatory benefit as artesunate. Interventions to promote hematological recovery and reduce the burden of malaria in the post-discharge period (e.g., post-discharge malaria chemoprevention) in high-risk children may confer additional protection against severe anemia [11, 54, 55].

Study strengths include the large study population; the detailed clinical characterization of study participants; the study design to assess behavioral and cognitive outcomes in children, which incorporated systematic assessment of factors related to child development such as socioeconomic status and enrichment in the home environment; and the use of previously validated instruments for assessment of cognition and behavior in this population, and the long-term follow-up of study participants. Study limitations included the observational nature of this trial, which included a change in standard medication for severe malaria over time, which could co-exist with other changes that might affect behavior. We adjusted for the most important of these, but unmeasured parameters that changed over time could also affect the outcomes in the study.

## Conclusions

In summary, the study confirms reduced mortality in children treated with artemisinin derivatives compared to quinine for the treatment of severe malaria. Further, we report better neurobehavioral and executive function in survivors treated with artesunate compared to quinine. The findings provide evidence that artesunate as compared to quinine therapy in children with severe malaria not only reduces mortality but also may provide a long-term benefit in behavioral and executive function in the survivors.

## Supplementary Information

**Additional file 1 **A .pdf file providing additional details on the Methods and Supplementary Tables and Figures. Methods: 1) Study population and clinical management of severe malaria. 2) Neurologic, cognitive and behavioral assessment. 3) Enrollment in the Iron study. **Supplementary Tables and Figures**: **Table S1**. Characteristics of severe malaria study participants by choice of in-hospital antimalarial administered**. Table S2**. Primary cognitive and behavioral outcomes in children over 24-month follow up, according to antimalarial treatment (quinine vs. parenteral artemisinin), and severe malaria group at presentation (cerebral malaria or severe malarial anemia)**. Table S3**. Primary cognitive and behavioral outcomes in children over 24-month follow up, according to specific antimalarial drug treatment, and severe malaria group at presentation (cerebral malaria or severe malarial anemia).

## Data Availability

The datasets used and/or analyzed during the current study are available from the senior author on reasonable request (email: chjohn@iu.edu).

## Abbreviations

AKI: Acute kidney injury
BRIEF: Behavior Rating Inventory of Executive Function
CBCL: Child Behavior Checklist
CC: Community children
CI: Confidence interval
CM: Cerebral malaria
COAT: Color Object Association Test
ECVT: Early Childhood Vigilance Test
EDTA: Ethylenediaminetetraacetic acid
FDR: False discovery rate
HOME: Home Observation for the Measurement of the Environment
HR: Hazards ratio
HRP-2: Histidine-rich protein-2
K-ABC: Kaufman Assessment Battery for Children
LDH: Lactate dehydrogenase
MSEL: Mullen Scales of Early Learning
OR: Odds ratio
SMA: Severe malarial anemia
TOVA: Test of Variables of Attention
