# Supplementary material for: Parenteral artemisinins are associated with reduced mortality and neurologic deficits and improved long-term behavioral outcomes in children with severe malaria
Source: BMC Med. 2021 Jul 28;19:168. doi: 10.1186/s12916-021-02033-1 (PMC8317420; doi:10.1186/s12916-021-02033-1)
Supplement: Supplementary file 1 — Additional file 1 A .pdf file providing additional details on the Methods and Supplementary Tables and Figures. Methods: 1) Study population and clinical management of severe malaria. 2) Neurologic, cognitive and behavioral assessment. 3) Enrollment in the Iron study. Supplementary Tables and Figures: Table S1. Characteristics of severe malaria study participants by choice of in-hospital antimalarial administered. Table S2. Primary cognitive and behavioral outcomes in children over 24-month follow up, according to antimalarial treatment (quinine vs. parenteral artemisinin), and severe malaria group at presentation (cerebral malaria or severe malarial anemia). Table S3. Primary cognitive and behavioral outcomes in children over 24-month follow up, according to specific antimalarial drug treatment, and severe malaria group at presentation (cerebral malaria or severe malarial anemia). [file 12916_2021_2033_MOESM1_ESM.docx]

**Additional File 1**

Supplement to: Conroy AL, Opoka RO, Bangirana, P, et al. Parenteral artemisinins are associated with reduced mortality and neurologic deficits and improved long-term behavioral outcomes in Ugandan children with severe malaria

[1) Methods 2](#_Toc73190102)

[**1)** **Study population and clinical management of severe malaria** 2](#_Toc73190103)

[**2)** **Neurologic, cognitive and behavioral assessment** 3](#_Toc73190104)

[**3)** **Enrollment in the Iron study** 4](#_Toc73190105)

[Supplementary Tables and Figures 5](#_Toc73190106)

[**Table S1. Characteristics of severe malaria study participants by choice of in-hospital antimalarial administered** 5](#_Toc73190107)

[**Table S2. Primary cognitive and behavioral outcomes in children over 24-month follow up, according to antimalarial treatment (quinine vs. parenteral artemisinin), and severe malaria group at presentation (cerebral malaria or severe malarial anemia)** 6](#_Toc73190108)

[**Table S3. Primary cognitive and behavioral outcomes in children over 24-month follow up, according to specific antimalarial drug treatment, and severe malaria group at presentation (cerebral malaria or severe malarial anemia)** 8](#_Toc73190109)

# **Methods**

## **Study population and clinical management of severe malaria**

All children with severe malaria had *P. falciparum* on blood smear. Children with cerebral malaria (CM) had a coma with no other identifiable cause ruling out meningitis, a prolonged postictal state, or hypoglycemia-associated coma reversed by a glucose infusion. Children with severe malarial anemia (SMA) had hemoglobin level ≤5g/dL. Exclusion criteria included known chronic illness requiring medical care, known developmental delay, or history of coma, head trauma, hospitalization for malnutrition, or cerebral palsy. Additional exclusion criteria in children with SMA included impaired consciousness on physical examination, other clinical evidence of central nervous system disease, or more than one seizure before admission. Additional exclusion criteria for the community controls (CC) included any illness requiring medical care within the previous four weeks or major medical or neurologic abnormalities at screening physical examination.

Children were managed according to the Uganda Clinical Guidelines at the time of the study. In the early phase of the study intravenous quinine hydrochloride was the first line treatment given as intravenous infusion of 10mg/kg in 5-10mL/kg of 5% glucose over a four hour period, repeated every eight hours until the child could take oral medication. The parenteral phase was followed by oral quinine 10mg/kg given three times as day to complete 7 days of treatment. Artemisinin-derivatives (artesunate or artemether) were second line therapy to be used if quinine was contraindicated or not available. In November 2012 updated Ugandan Clinical Guidelines were published recommending parenteral artesunate as the first line antimalarial for severe malaria (2.4mg/kg for children >20kg or 3.0mg/kg for children <20kg given at time 0, 12, and 24 hours, and daily until the child could tolerate oral medication. The oral treatment was a three day course of artemether/lumefantrine. The change to artemisinin derivatives was implemented gradually in the health units from 2012 depending on the availability of the medicines. Hypoglycemia was treated with a 1-2mL/kg 25% dextrose bolus administered intravenously. Fluid resuscitation was managed conservatively according to local guidelines at the time of the study: a fluid bolus of 20 ml/kg of sodium chloride 0.9% intravenously over 1 hour was given only for treatment of shock (systolic blood pressure<50mmHg or absent peripheral pulse) with delayed capillary refill (>2 seconds). Children without shock but with evidence of dehydration received maintenance intravenous fluids. All children underwent a medical history and physical examination on enrollment. An assessment of enrichment in the home environment and guardian interaction with the child was conducted at six months follow-up by a trained home visitor using age-appropriate versions of the Home Observation for the Measurement of the Environment (HOME)[9].

## **Neurologic, cognitive and behavioral assessment**

Neurologic status was assessed by a study clinician at discharge, 6 months, and at one and two years follow-up. Neurologic deficits were assessed through a standardized neurologic exam conducted by a study physician. A neurologic deficit was defined as speech difficulties including aphasia or speech regression, visual impairment or blindness, motor deficits (hypertonia, hypotonia, spasticity, cranial nerve palsy), movement disorders (tremor, dystonia, choreoathetoid), ataxia, hearing deficits, hyporeflexia, or Babinski sign. Cognitive and behavioral assessments were conducted by trained neuropsychological testers without knowledge of the child’s disease history. Cognitive evaluations were conducted by assessors with demonstrated proficiency in testing. The Mullen Scales of Early Learning (MSEL) were used to measure cognitive ability in children less than five years of age[21]. Scores from fine motor, visual reception, receptive language and expressive language scales were summed to give the early learning composite score as a measure of overall cognitive ability. Associative memory was assessed using the Color Object Association Test (COAT), in which children are required to associate toys with specific color coded boxes and scored on the total number of toys placed in the correct boxes[22]. Attention was assessed using the Early Childhood Vigilance Test (ECVT)[23], in which a child was required to focus his/her gaze on cartoons screened on a computer for about 7 minutes. The measure of attention is the percent time the child spent gazing at the screen. In children 5 years and older, the Kaufman Assessment Battery for Children (K-ABC second edition) was used to measure overall cognitive ability[24]. Laura’s model was used to obtain a composite score including sequential processing, simultaneous processing, learning ability and planning ability. Attention in these children was assessed using the Test of Variables of Attention (TOVA)[25] to measure attention and impulse control with D-prime as the primary measure of attention. Memory in children >5 years of age is the score for sequential processing. Socio-emotional function was assessing using the preschool (18 months-6 years) and school-aged (6-12 years) Child Behavioral Checklist (CBCL)[26], and executive function with the preschool (2-6 years) and school-aged (6-12 years) Behavior Rating Inventory of Executive Function (BRIEF)[27]. Higher z-scores on CBCL and BRIEF indicate poorer performance and more problematic behavior, while higher z-scores in cognition, attention, memory indicate better performance.

Age-adjusted z-scores were created using the scores of the community children (CC)[9]. For each outcome, the z-score was computed as (actual score minus average score for child's age)/SD, where "average score for child's age" and "SD" were computed by fitting a mixed linear model to data from all available visits for CC (allowing correlated errors for a child's multiple visits). Z-scores have average of 0 and SD 1 in the reference population (CC) over all time points. Age-adjusted z-scores for cognition, attention, memory, and behavior (socio-emotional function and executive function) were generated separately for children stratified by age (<5 or ≥5 for cognition, attention, memory; <6 or ≥6 for behavior, based on the test-specified age cut-offs).

## **Enrollment in the Iron study**

In June 2010 a clinical trial evaluating immediate versus delayed iron therapy recruited within the study cohort where children with a zinc protoporphyrin concentration >80μmol/mol heme were randomized to 27-day ferrous sulfate at 2mg/kg/day concurrently with antimalarial therapy or delayed iron therapy on day 28. The acute vs. delayed iron clinical trial sub-component of this research study was registered at clinicaltrials.gov as NCT01093989. Children were visited at home by study home visitors every two weeks, starting two weeks after enrollment and continuing until month 4, by which time all children had finished iron therapy. Home visits were made monthly thereafter. Home visitors measured each child’s temperature and referred any ill child to the study clinic for care. They also assessed home environment (16), medication adherence, and side effects of iron treatment. Parents were asked to bring the child to the study clinic or hospital for any illness.

# **Supplementary Tables and Figures**

### **Table S1. Characteristics of severe malaria study participants by choice of in-hospital antimalarial administered**

|  | **Antimalarial medication** | | | | |
| --- | --- | --- | --- | --- | --- |
|  | **Quinine alone**  **(n=346)** | **Artemether and Quinine (n=74)** | **Artemether alone (n=31)** | **Artesunate (n=51)** | **P** |
| Age, years | 3.7 (1.9) | 3.7 (2.0) | 3.9 (1.4) | 3.6 (1.6) | 0.939 |
| Sex, % (F) | 142 (41.0) | 25 (33.8) | 11 (35.5) | 25 (49.0) | 0.349 |
| Weight-for-age z score | -1.2 (1.1) | -1.1 (1.0) | -0.8 (0.9) | -1.0 (1.4) | 0.336 |
| Height-for-age z score | -1.2 (1.4) | -1.2 (1.4) | -1.1 (1.4) | -1.7 (1.4) | 0.067 |
| Duration of fever, history | 3.8 (2.7) | 3.4 (1.6) | 3.2 (2.0) | 4.8 (2.7) | 0.011 |
| Socioeconomic status score^1^ | 9.4 (3.0) | 9.6 (3.3) | 9.4 (3.1) | 10.3 (3.7) | 0.341 |
| Home environment z score^1^ | -0.10 (0.98) | 0.05 (1.1) | -0.02 (0.99) | 0.32 (0.99) | 0.056 |
| Maternal education, No. (%)^1^  Primary 6 or lower  Primary 7  Secondary or higher  Not known | 121 (38.2)  69 (21.8)  107 (33.8)  20 (6.3) | 22 (31.4)  12 (17.1)  34 (48.6)  2 (2.9) | 9 (30.0)  7 (23.3)  13 (43.3)  1 (3.3) | 24 (48.9)  8 (16.3)  15 (30.6)  2 (4.1) | 0.344 |
| Paternal education, No. (%)^1^  Primary 6 or lower  Primary 7  Secondary or higher  Not known | 61 (19.2)  50 (15.8)  130 (41.0)  76 (24.0) | 13 (18.6)  8 (11.4)  40 (57.1)  9 (12.9) | 6 (20.0)  7 (23.3)  15 (50.0)  2 (6.7) | 16 (32.7)  10 (20.4)  17 (34.7)  6 (12.2) | 0.021 |
| Child education, No. (%)^1^ | 92 (30.1) | 20 (29.0) | 12 (40.0) | 20 (42.6) | 0.250 |
| Severe malaria group, No (%)  Cerebral malaria  SMA | 186 (69.1)  160 (68.7) | 51 (19.0)  23 (9.9) | 12 (4.5)  19 (8.2) | 20 (7.4)  31 (13.3) | 0.003 |
| **Laboratory characteristics^2^** |  |  |  |  |  |
| Hemoglobin, g/dL | 4.8 (3.9, 7.2) | 5.2 (4.1, 6.9) | 4.5 (3.3, 5.1) | 4.4 (3.3, 6.1) | 0.037 |
| Glucose, mmol/L | 6.3 (4.8, 8.5) | 6.4 (5.0, 8.7) | 6.8 (4.2, 7.8) | 7.1 (4.6, 9.0) | 0.824 |
| Lactate, mmol/L | 3.8 (2.2, 6.6) | 5.1 (2.8, 8.4) | 4.5 (3.0, 8.0) | 6.3 (3.9, 8.2) | 0.0005 |
| WBC, x10^3^/μL | 10.2 (7.3, 15.1) | 10.6 (8.0, 13.4) | 10.3 (8.1, 17.4) | 11.7 (7.7, 17.4) | 0.622 |
| Platelet, x10^3^/μL | 103 (51, 176) | 78 (35, 144) | 87 (51, 206) | 104 (41, 200) | 0.124 |
| Lactate dehydrogenase, U/L | 762 (608, 1058) | 859 (680, 1142) | 990 (769, 1780) | 742 (632, 929) | 0.0008 |
| Parasite density, /uL | 45280 (11400, 209460) | 48050 (14810, 323060) | 17640 (6220, 68300) | 35740 (1700, 111020) | 0.014 |
| Plasma PfHRP2, ng/mL | 1654 (563, 3638) | 2856 (987, 5702) | 1294 (350, 6684) | 2958 (469, 5675) | 0.008 |
| Creatinine, mg/dL | 0.38 (0.30, 0.49) | 0.39 (0.29, 0.51) | 0.47 (0.33, 0.57) | 0.34 (0.27, 0.44) | 0.017 |
| BUN, mg/dL | 14 (10, 21) | 16 (13, 23) | 21 (14, 32) | 12 (8, 20) | 0.0009 |
| **Clinical complications and recovery** | | | | | |
| Parasite clearance time | 2 (1, 3) | 2 (2, 3) | 2 (1, 2) | 1 (1, 2) | 0.0006 |
| Coma duration^2^ | 48 (29, 80) | 54 (40, 82) | 75 (39, 90) | 54 (44, 110) | 0.035 |
| Seizure no. in hospital^2^ | 1 (0, 2) | 1 (0, 2) | 1 (0, 2) | 1 (0, 3) | 0.712 |
| Hypoglycemia, No (%) | 26 (7.5) | 7 (9.5) | 2 (6.5) | 1 (2.0) | 0.432 |
| **Co-treatments** | | | | | |
| Dextrose bolus, No (%) | 203 (58.7) | 52 (70.3) | 15 (48.4) | 22 (43.1) | 0.015 |
| Transfusion, No (%) | 254 (73.4) | 58 (78.4) | 30 (96.8) | 45 (88.2) | 0.004 |
| IV fluids, No (%) | 52 (15.0) | 14 (18.9) | 7 (22.6) | 5 (9.8) | 0.367 |
| Furosemide, No (%) | 71 (20.5) | 10 (13.5) | 3 (9.7) | 3 (5.9) | 0.026 |
| Antibiotics, No (%) | 174 (50.3) | 42 (56.8) | 17 (54.8) | 31 (60.8) | 0.443 |
| Enrolled in Iron study, No (%) | 77 (22.3) | 29 (39.2) | 12 (38.7) | 38 (74.5) | <0.001 |

### **Table S2. Primary cognitive and behavioral outcomes in children over 24-month follow up, according to antimalarial treatment (quinine vs. parenteral artemisinin), and severe malaria group at presentation (cerebral malaria or severe malarial anemia)**

|  | **Preschool age at testing** | | | | **School age at testing** | | | |
| --- | --- | --- | --- | --- | --- | --- | --- | --- |
|  | **N (obs), N ^a^** | **Mean difference ^b^,**  **artemisinin derivatives vs. quinine (95% CI)** | **P value** | **Sig.**  **†*** | **N (obs), N ^a^** | **Mean difference ^b^,**  **artemisinin derivatives vs. quinine (95% CI)** | **P value** | **Sig.**  **†*** |
| **Cerebral malaria** | | | | | | | | |
| **Cognition Outcomes ^c^** | **<5 years of age** | | | | **≥ 5 years of age** | | | |
| Cognition | 505, 171 | -0.21 (-0.71, 0.29) | 0.402 |  | 341, 148 | 0.03 (-0.61, 0.68) | 0.922 |  |
| Attention | 527, 171 | 0.14 (-0.16, 0.45) | 0.350 |  | 341, 148 | 0.14 (-0.36, 0.63) | 0.590 |  |
| Memory | 526, 172 | -0.09 (-0.29, 0.10) | 0.337 |  | 344, 148 | 0.24 (-0.31, 0.79) | 0.395 |  |
| **Behavioral Outcomes ^d^** | **<6 years of age** | | | | **≥ 6 years of age** | | | |
| **Child Behavior Checklist** |  |  |  |  |  |  |  |  |
| Internalizing behavior | 656, 189 | -0.20 (-0.46, 0.06) | 0.132 |  | 219, 86 | -0.43 (-0.85, -0.01) | 0.047 | † |
| Externalizing behavior | 656, 189 | -0.32 (-0.59, -0.05) | 0.019 | † | 219, 86 | -0.20 (-0.85, 0.45) | 0.545 |  |
| Total behavior | 656, 189 | -0.34 (-0.63, -0.04) | 0.024 | † | 219, 86 | -0.32 (-0.95, 0.30) | 0.303 |  |
| **Behavior Rating Inventory** |  |  |  |  |  |  |  |  |
| Global Executive Composite | 363, 149 | -0.83 (-1.39, -0.27) | 0.004 | †* | 153, 81 | -0.28 (-0.74, 0.18) | 0.236 |  |
| **Severe malarial anemia** | | | | | | | | |
| **Cognition Outcomes ^c^** | **<5 years of age** | | | | **≥ 5 years of age** | | | |
| Cognition | 548, 180 | 0.17 (-0.30, 0.64) | 0.478 |  | 243, 100 | -0.04 (-0.75, 0.66) | 0.904 |  |
| Attention | 584, 181 | -0.08 (-0.36, 0.20) | 0.568 |  | 249, 103 | 0.26 (-0.36, 0.89) | 0.400 |  |
| Memory | 565, 178 | -0.06 (-0.29, 0.17) | 0.609 |  | 250, 103 | 0.08 (-0.53, 0.69) | 0.791 |  |
| **Behavioral Outcomes ^d^** | **<6 years of age** | | | | **≥ 6 years of age** | | | |
| **Child Behavior Checklist** |  |  |  |  |  |  |  |  |
| Internalizing behavior | 683, 201 | -0.58 (-0.88, -0.29) | 0.0001 | †* | 134, 66 | -0.25 (-0.80, 0.31) | 0.378 |  |
| Externalizing behavior | 683, 201 | -0.34 (-0.64, -0.04) | 0.024 | †* | 134, 66 | -0.49 (-1.33, 0.34) | 0.241 |  |
| Total behavior | 683, 201 | -0.48 (-0.81, -0.15) | 0.004 | †* | 134, 66 | -0.62 (-1.36, 0.11) | 0.092 |  |
| **Behavior Rating Inventory** |  |  |  |  |  |  |  |  |
| Global Executive Composite | 372, 157 | -0.75 (-1.25, -0.25) | 0.004 | †* | 94, 58 | -0.54 (-1.15, 0.07) | 0.080 |  |

^a^ N (obs) refers to the number of observations of cognitive or behavioral measures included in the model and N refers to the number of study participants.

^b^ Mean difference and 95% confidence interval (CI) are derived from a linear mixed effects model, with the beta coefficient representing the mean difference. Models included a subject specific random intercept and random caretaker effect, time as a categorical variable (to allow for non-linearity between study visits), and adjusted for age, sex, height-for-age and weight-for-age z score, socioeconomic status and home environment z score, disease severity during the acute illness (number of seizures, coma, acute kidney injury), child schooling, enrollment in the iron study, and the number of admissions over 24 months follow-up.

^c^ For cognitive outcomes a positive number is indicative of an improved outcome in children receiving artemisinin-derivatives. In preschool aged children (<5 years of age), cognition was assessed using the Mullen Scales of Early Learning, attention was assessed using the early childhood vigilance test, and memory was assessed using the color object association test. In school aged children (≥5 years of age), cognition was assessed using the Kauffman Assessment Battery for Children (K-ABC) second edition using the mental processing index, attention was assessed using the Test of Variables of Attention using the D-prime measure, and memory was assessed using the sequential processing from K-ABC.

^d^ For behavioral outcomes a negative number is indicative of an improved outcome in children receiving artemisinin-derivatives. †p<0.05, *adjusted p<0.05 Benjamini-Hoch correction for False Discovery Rate at 0.05 adjusting for 7 comparisons within each age strata and study group (cerebral malaria vs. severe malarial anemia).

### **Table S3. Primary cognitive and behavioral outcomes in children over 24-month follow up, according to specific antimalarial drug treatment, and severe malaria group at presentation (cerebral malaria or severe malarial anemia)**

|  | **Preschool age at testing** | | | | **School age at testing** | | | |
| --- | --- | --- | --- | --- | --- | --- | --- | --- |
|  | **N (obs), N ^a^** | **Mean difference ^b^,**  **artemisinin derivatives vs. quinine (95% CI)** | **P value** | **Sig.**  **†*** | **N (obs), N ^a^** | **Mean difference ^b^,**  **artemisinin derivatives vs. quinine (95% CI)** | **P value** | **Sig.**  **†*** |
| **Cerebral malaria** |  |  |  |  |  |  |  |  |
| **Cognition Outcomes ^c^** | **<5 years of age** | | | | **≥ 5 years of age** | | | |
| Cognition  Quinine + Artemether  Artemether  Artesunate | 505, 171 | -0.20 (-0.76, 0.36)  -0.31 (-1.46, 0.84)  -0.20 (-1.18, 0.78) | 0.484  0.594  0.690 |  | 341, 148 | -0.02 (-0.77, 0.72)  0.24 (-1.33, 1.80)  0.08 (-1.06, .123) | 0.951  0.766  0.884 |  |
| Attention  Quinine + Artemether  Artemether  Artesunate | 527, 171 | 0.20 (-0.13, 0.54)  -0.16 (-0.88, 0.57)  0.09 (-0.52, 0.71) | 0.234  0.668  0.771 |  | 341, 148 | 0.07 (-0.50, 0.65)  0.37 (-0.83, 1.57)  0.19 (-0.69, 1.07) | 0.800  0.540  0.671 |  |
| Memory  Quinine + Artemether  Artemether  Artesunate | 526, 172 | -0.07 (-0.29, 0.14)  -0.06 (-0.51, 0.14)  -0.22 (-0.60, 0.16) | 0.512  0.774  0.258 |  | 344, 148 | 0.25 (-0.39, 0.89)  -0.29 (-1.62, 1.05)  0.46 (-0.51, 1.44) | 0.436  0.672  0.348 |  |
| **Behavioral Outcomes ^d^** | **<6 years of age** | | | | **≥ 6 years of age** | | | |
| **Child Behavior Checklist** |  |  |  |  |  |  |  |  |
| Internalizing behavior  Quinine + Artemether  Artemether  Artesunate | 656, 189 | -0.15 (-0.44, 0.14)  -0.19 (-0.79, 0.40)  -0.43 (-0.95, 0.09) | 0.314  0.517  0.107 |  | 219, 86 | -0.18 (-0.70, 0.34)  -0.78 (-1.67, 0.11)  -0.72 (-1.40, -0.04) | 0.495  0.085  0.039 |  |
| Externalizing behavior  Quinine + Artemether  Artemether  Artesunate | 656, 189 | -0.24 (-0.54, 0.06)  -0.55 (-1.14, 0.04)  -0.48 (-1.01, 0.06) | 0.111  0.068  0.081 |  | 219, 86 | -0.11 (-0.91, 0.68)  -0.17 (-1.56, 1.22)  -0.40 (-1.48, 0.67) | 0.782  0.809  0.455 |  |
| Total behavior  Quinine + Artemether  Artemether  Artesunate | 656, 189 | -0.29 (-0.61, 0.04)  -0.49 (-1.14, 0.17)  -0.43 (-1.02, 0.15) | 0.086  0.142  0.146 |  | 219, 86 | -0.06 (-0.82, 0.70)  -0.44 (-1.75, 0.87)  -0.83 (-1.85, 0.18) | 0.871  0.504  0.107 |  |
| **Behavior Rating Inventory** |  |  |  |  |  |  |  |  |
| Global Executive Composite  Quinine + Artemether  Artemether  Artesunate | 363, 149 | -0.59 (-1.22, 0.04)  -1.50 (-2.87, -0.13)  -1.25 (-2.23, -0.28) | 0.064  0.032  0.012 | †  † | 153, 81 | -0.09 (-0.64, 0.46)  -0.33 (-1.30, 0.64)  -0.64 (-1.37, 0.09) | 0.742  0.498  0.084 |  |
| **Severe malarial anemia** |  |  |  |  |  |  |  |  |
| **Cognition Outcomes ^c^** | **<5 years of age** | | | | **≥ 5 years of age** | | | |
| Cognition  Quinine + Artemether  Artemether  Artesunate | 548, 180 | - 1. (-0.60, 0.62)   0.83 (0.06, 1.61)  -0.05 (-0.71, 0.62) | 0.965  0.036  0.892 | † | 243, 100 | 0.23 (-0.94, 1.40)  0.37 (-0.60, 1.34)  -0.60 (-1.54, 0.36) | 0.697  0.451  0.220 |  |
| Attention  Quinine + Artemether  Artemether  Artesunate | 584, 181 | -0.12 (-0.48, 0.24)  0.11 (-0.37, 0.59)  -0.14 (-0.54, 0.26) | 0.502  0.663  0.485 |  | 249, 103 | 0.23 (-0.82, 1.27)  0.51 (-0.35, 1.38)  0.05 (-0.80, 0.89) | 0.668  0.241  0.912 |  |
| Memory  Quinine + Artemether  Artemether  Artesunate | 565, 178 | -0.05 (-0.35, 0.26)  0.16 (-0.23, 0.54)  -0.22 (-0.55, 0.11) | 0.752  0.432  0.198 |  | 250, 103 | 0.09 (-0.93, 1.11)  0.34 (-0.51, 1.19)  -0.17 (-0.99, 0.66) | 0.862  0.432  0.692 |  |
| **Behavioral Outcomes ^d^** | **<6 years of age** | | | | **≥ 6 years of age** | | | |
| **Child Behavior Checklist** |  |  |  |  |  |  |  |  |
| Internalizing behavior  Quinine + Artemether  Artemether  Artesunate | 683, 201 | -0.27 (-0.66, 0.11)  -0.57 (-1.06, -0.09)  -0.95 (-2.35, -0.55) | 0.166  0.020  <0.0001 | †  †* | 134, 66 | -0.47 (-1.43, 0.49)  0.15 (-0.62, 0.91)  -0.54 (-1.32, 0.25) | 0.325  0.701  0.177 |  |
| Externalizing behavior  Quinine + Artemether  Artemether  Artesunate | 683, 201 | 0.10 (-0.29, 0.49)  -0.47 (-0.95, 0.01)  -0.79 (-1.19, -0.38) | 0.606  0.056  0.0002 | †* | 134, 66 | -0.10 (-1.38, 1.20)  0.41 (-0.61, 1.42)  -1.84 (-2.90, -0.79) | 0.888  0.427  0.001 | †* |
| Total behavior  Quinine + Artemether  Artemether  Artesunate | 683, 201 | -0.11 (-0.54, 0.33)  -0.46 (-0.99, 0.07)  -0.94 (-1,38, -0.49) | 0.628  0.091  0.0001 | †* | 134, 66 | -0.63 (-1.79, 0.54)  0.36 (-0.57, 1.29)  -1.63 (-2.59, -0.67) | 0.278  0.433  0.002 | †* |
| **Behavior Rating Inventory** |  |  |  |  |  |  |  |  |
| Global Executive Composite  Quinine + Artemether  Artemether  Artesunate | 372, 157 | -0.26 (-0.92, 0.41)  -0.34 (-1.13, 0.46)  -1.38 (-2.00, -0.76) | 0.450  0.403  <0.0001 | †* | 94, 58 | -0.48 (-1.50, 0.54)  -0.12 (-0.89, 0.65)  -1.14 (-1.94, -0.33) | 0.344  0.759  0.007 | † |

^a^ N (obs) refers to the number of observations of cognitive or behavioral measures included in the model and N refers to the number of study participants.

^b^ Mean difference and 95% confidence interval (CI) are derived from a linear mixed effects model, with the beta coefficient representing the mean difference. Models included a subject specific random intercept and random caretaker effect, time as a categorical variable (to allow for non-linearity between study visits), and adjusted for age, sex, height-for-age and weight-for-age z score, socioeconomic status and home environment z score, disease severity during the acute illness (number of seizures, coma, acute kidney injury), child schooling, enrollment in the iron study, and the number of admissions over 24 months follow-up.

^c^ For cognitive outcomes a positive number is indicative of an improved outcome in children receiving artemisinin-derivatives. In preschool aged children (<5 years of age), cognition was assessed using the Mullen Scales of Early Learning, attention was assessed using the early childhood vigilance test, and memory was assessed using the color object association test. In school aged children (≥5 years of age), cognition was assessed using the Kauffman Assessment Battery for Children (K-ABC) second edition using the mental processing index, attention was assessed using the Test of Variables of Attention using the D-prime measure, and memory was assessed using the sequential processing from K-ABC.

^d^ For behavioral outcomes a negative number is indicative of an improved outcome in children receiving artemisinin-derivatives.

†p<0.05, *adjusted p<0.05 Benjamini-Hoch correction for False Discovery Rate at 0.05 adjusting for 7 comparisons within each age strata and study group (cerebral malaria vs. severe malarial anemia).
